# Bleeding Risk During Colorectal Endoscopic Mucosal Resection With Continued Anticoagulant Therapy: A Multicenter Study

**DOI:** 10.7759/cureus.76753

**Published:** 2025-01-01

**Authors:** Sayo Kobayashi, Keita Harada, Toru Nawa, Tomoo Fujisawa, Toru Ueki, Junichiro Nasu, Yuki Morito, Tatsuya Toyokawa, Tomoki Inaba, Masahide Kita, Ryuta Takenaka, Masafumi Inoue, Reiji Higashi, Takao Tsuduki, Minoru Matsubara, Yasushi Yamasaki, Hiroyuki Okada, Motoyuki Otsuka

**Affiliations:** 1 Department of Internal Medicine, Fukuyama City Hospital, Fukuyama, JPN; 2 Department of Gastroenterology, Okayama University Hospital, Okayama, JPN; 3 Department of Gastroenterology, Okayama Saiseikai General Hospital, Okayama, JPN; 4 Department of Internal Medicine, Mitoyo General Hospital, Kanonji, JPN; 5 Department of Internal Medicine, Hiroshima City Hiroshima Citizens Hospital, Hiroshima, JPN; 6 Department of Gastroenterology, Fukuyama Medical Center, Fukuyama, JPN; 7 Department of Gastroenterology, Kagawa Prefectural Central Hospital, Takamatsu, JPN; 8 Department of Gastroenterology, Okayama City Civic Hospital, Okayama, JPN; 9 Department of Internal Medicine, Tsuyama Chuo Hospital, Tsuyama, JPN; 10 Department of Gastroenterology, Japanese Red Cross Okayama Hospital, Okayama, JPN; 11 Department of Gastroenterology, Ichinomiya Nishi Hospital, Ichinomiya, JPN; 12 Department of Internal Medicine, Japanese Red Cross Society Himeji Hospital, Himeji, JPN; 13 Department of Gastroenterology, Sumitomo Besshi Hospital, Niihama, JPN

**Keywords:** anticoagulant therapy, direct oral anticoagulant, endoscopic mucosal dissection, endoscopic mucosal resection, thrombosis, warfarin

## Abstract

Background and aims

Endoscopic mucosal resection (EMR) is a standard preventive method for colorectal cancer. Managing patients on anticoagulants during EMR is challenging because of balancing bleeding and thrombotic risks. Updated guidelines recommend continuing anticoagulants over heparin bridging; however, data on bleeding risks with continuing anticoagulants remain limited. This multicenter prospective study evaluated bleeding rates in patients who continued oral anticoagulants during EMR.

Methods

Patients on warfarin or direct oral anticoagulants (DOACs) undergoing EMR were enrolled from 12 tertiary hospitals. Warfarin was maintained on the day of EMR, while DOACs were paused on the day of EMR and resumed afterward. Post-EMR mucosal defects were closed with clips per protocol. Adverse events were monitored for 30 days. The primary endpoint was the major bleeding rate, defined as immediate bleeding requiring difficult hemostasis or delayed bleeding necessitating endoscopic intervention. The secondary endpoints were minor bleeding, other adverse events, and differences in bleeding rates between warfarin and DOACs.

Results

Among 107 patients (341 polyps; mean size = 6.7 mm), major bleeding occurred in five (4.7%) patients (95% confidence interval: 2.0%-10.5%), and all cases were managed endoscopically. Minor bleeding and thromboembolism events occurred in eight (7.5%) patients and one (0.9%) patient, respectively. No significant differences in bleeding rates were observed between warfarin and DOACs. Major bleeding rates were lower than those reported for heparin bridging.

Conclusions

Continuing anticoagulant therapy during EMR was associated with a low major bleeding rate (4.7%) and minimal thrombotic events, supporting its safety as an alternative to heparin bridging.

## Introduction

Colorectal cancer (CRC) is the third most common cancer globally and the second leading cause of cancer-related deaths worldwide [1]. Endoscopic polypectomy of colorectal polyps is the standard preventive method for CRC, with well-established efficacy in reducing its incidence [2-5].

As the population with ages and indications for anticoagulation therapy expands, the number of patients receiving anticoagulants continues to grow [6]. Polypectomy in patients on anticoagulants increases the risk of peri-procedural bleeding [7,8], while cessation of anticoagulants raises the risk of thrombotic events. Previous guidelines recommended replacing anticoagulants with heparin during endoscopic polypectomy [9]; however, heparin replacement is associated with a high rate of post-polypectomy bleeding [10-12]. Updated guidelines have recently discouraged heparin replacement for patients undergoing polypectomy while on anticoagulants. Instead, they propose continuing warfarin within the therapeutic range or continuing direct oral anticoagulants (DOACs) until the day before the procedure [13].

However, the supporting data for this updated guideline were limited. In particular, there was a lack of data focusing on peri-procedural bleeding rates in patients who continued anticoagulant therapy during endoscopic polypectomy. To address this gap, we conducted a multicenter, prospective, observational study to assess peri-polypectomy bleeding rates associated with continuous anticoagulant use.

## Materials and methods

Study design

This prospective interventional study was conducted across 12 tertiary hospitals. The study protocol (No. 1611-004) received approval from the Institutional Review Board (IRB) of Okayama University Hospital and the IRBs of the other participating hospitals. The study was registered in the University Hospital Medical Information Network Clinical Trials Registry (UMIN) under the identifier UMIN 00021416. Written informed consent was obtained from all patients in accordance with the Declaration of Helsinki.

Patient enrollment began after IRB approval and study registration. Patients were provided with a detailed explanation of the study objectives, the polypectomy procedure, the anticipated risks, and the continuation of anticoagulant therapy. Written informed consent was then obtained from all participants. Relevant data, including the indication for anticoagulant therapy, type of anticoagulant agents, procedure outcomes, and adverse events, were prospectively collected and documented in case report forms. These forms were promptly submitted to the data center at Okayama University Hospital. The study adhered to the Strengthening the Reporting of Observational Studies in Epidemiology (STROBE) guidelines. All authors had access to the study data and reviewed and approved the final manuscript.

Participants

Patients with previously identified colorectal polyps who were candidates for endoscopic mucosal resection (EMR) were assessed for eligibility. The inclusion criteria were a lesion size of <20 mm as determined by endoscopic evaluation, monotherapy with warfarin or a DOAC as the antithrombotic therapy, therapeutic range of warfarin (prothrombin time-international normalized ratio of <2.6), platelet count of ≥100,000/mm^3^, age of ≥18 years, and provision of written informed consent.

The exclusion criteria were the use of antiplatelet agents on the day of EMR, use of steroids, severe organ failure, hemodialysis, and unsuitability for participation in the study. Patients receiving dual therapy with an anticoagulant and an antiplatelet agent were eligible for inclusion if they had a low risk of thrombosis and the antiplatelet agent could be discontinued one week prior to EMR, in accordance with the established guidelines [13].

**Figure 1:**
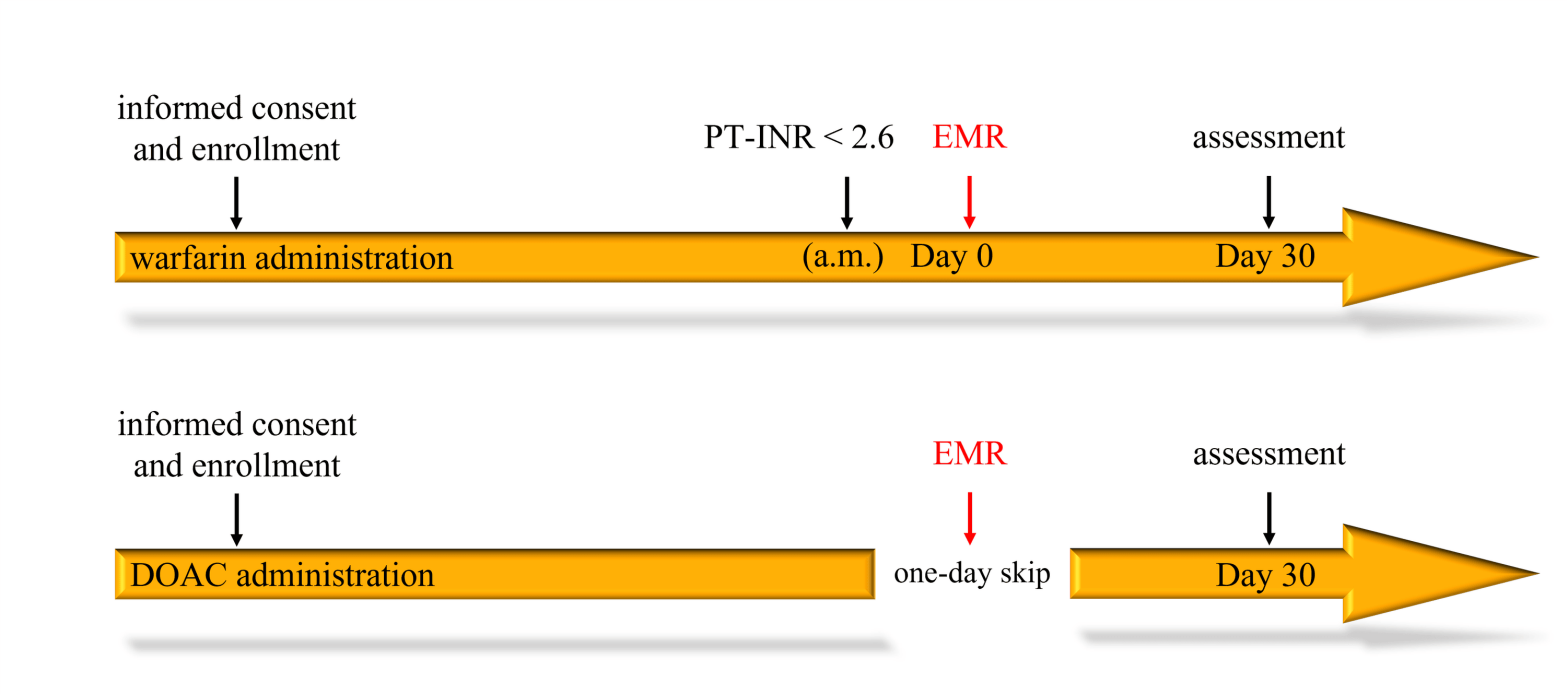
Protocol for continued anticoagulants. Warfarin was maintained on the day of EMR, while DOACs were discontinued on the day of EMR and resumed the following day. EMR: endoscopic mucosal resection; DOACs: direct oral anticoagulants; PT-INR: prothrombin time-international normalized ratio.

Patients who met the inclusion criteria and were scheduled for EMR with continued anticoagulant therapy were enrolled prior to the procedure. The anticoagulant already taken was continued. Warfarin was maintained on the day of the procedure, while DOACs were discontinued on the day of the procedure and resumed afterward (Figure 1). All lesions meeting the eligibility criteria, except for serrated lesions size of <5 mm, were resected by EMR.

EMR procedure

All procedures were performed using standard colonoscopes (PCF-Q260AZI or PCF-H290ZI (Olympus, Tokyo, Japan) or EC-L600ZP (Fujifilm Co., Tokyo, Japan)). A polypectomy snare ranging in size from 10 to 20 mm was selected based on the size of the polyp. After advancing the scope to the lesion, the EMR procedure was begun.

The lesion was carefully delineated using white light endoscopy. If the lesion margins were not clearly visible under white light, chromoendoscopy with indigo carmine was used. Following lesion evaluation, physiological saline was injected into the submucosa. A polypectomy snare was deployed to capture the polyp along with the surrounding mucosa, and the tissue was resected using electrical current (e.g., at Okayama University Hospital, the VIO 300D (ERBE Elektromedizin, Tübingen, Germany) was used in Endo Cut Q mode with the following settings: effect = 3, duration = 2, interval = 4, and forced coagulation, effect = 2, 40 W).

After resection, the mucosal defect was carefully inspected, and immediate bleeding was evaluated. In cases of immediate bleeding, endoscopic hemostasis was performed using techniques such as clipping or coagulation with hemostatic forceps. Closure of mucosal defect with conventional clips was subsequently carried out in all cases, as a protocol procedure. Specimens obtained through EMR were fixed in 10% formalin and assessed by expert pathologists at each participating institution.

Follow-up after EMR

An outpatient medical check-up or telephone interview was conducted 30 days after the EMR to monitor for any delayed adverse events.

Endpoints and definitions

The primary endpoint was the rate of major bleeding occurring within 30 days following EMR. Major bleeding was defined as active immediate bleeding that was difficult to control endoscopically, delayed bleeding resulting in hematochezia, or a ≥2 g/dL reduction in hemoglobin requiring endoscopic hemostasis.

The secondary endpoints were the rate of minor bleeding, the rate of thrombotic events, and the difference in bleeding rates between warfarin and DOAC therapy. Minor bleeding was defined as hematochezia that did not require endoscopic hemostasis and was managed with conservative measures, achieving spontaneous hemostasis.

Sample sizes and statistical assessment

The reported rate of major bleeding in patients undergoing heparin replacement during EMR is 24% [14], while the rate in patients continuing warfarin during EMR is 14% [15]. In the report of continuing warfarin during EMR, clip closure of the mucosal defect was not prescribed. While, in this study, clip closure of the defect was included as part of the protocol. Based on this information, the expected rate of major bleeding was set at 10%, with a threshold of 20%. To achieve 80% power with a two-sided α of 5%, the required sample size was calculated to be 82 lesions. To account for a 10% dropout rate, the enrollment target was set at 90 lesions.

Results are presented as mean ± standard deviation for continuous variables. For the rate of major bleeding, the 95% confidence interval (CI) was calculated using the exact CI based on a binomial distribution. Fisher’s exact test was used to compare categorical data, with a p-value of <0.05 considered statistically significant. Analysis was performed using JMP 17 software (SAS Institute, Cary, NC, USA).

## Results

Participants and characteristics

**Figure 2:**
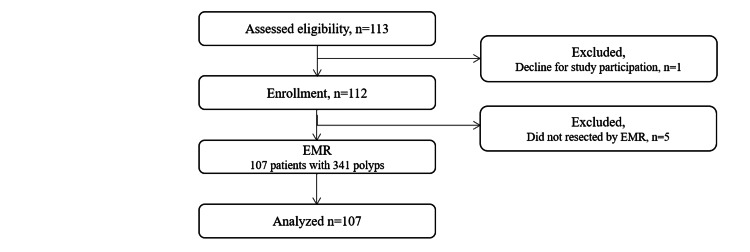
Patient flowchart. EMR: endoscopic mucosal resection.

From February 2015 to December 2017, 113 patients were assessed for eligibility, with one patient excluded due to the decision to decline participation in the study. Of the remaining 112 patients enrolled, five were excluded because no polyps were resected via EMR. Consequently, 107 patients were analyzed in this study and 341 polyps were removed (Figure 2). The baseline characteristics of the patients are summarized in Table 1. Warfarin was administered to 56 (52.3%) patients, while DOACs were used in 51 (47.7%). Dual antiplatelet therapy was administered to 13 (12.1%), with all antiplatelet agents discontinued one week prior to EMR. Most patients (74.8%) were on anticoagulant therapy primarily for atrial fibrillation management.

**Table 1:** Patients’ baseline characteristics. Data are presented as mean ± standard deviation or n (%). * Some patients had several indications for antithrombotic therapy. DOAC: direct oral anticoagulant; PT: prothrombin time-international normalized ratio.

	n = 107
Age, years	72.7 ± 7.8
Male/female	84/22
Body mass index, kg/m^2^	23.8 ± 3.5
Anticoagulant	
Warfarin	56 (52.3)
DOAC	51 (47.7)
Dual therapy with antiplatelet	13 (12.1)
Aspirin	9 (8.4)
Thienopyridine	2 (1.9)
Cilostazol	1 (0.9)
Indications for antithrombotic therapy*	
Atrial fibrillation	80 (74.8)
Valvular heart disease	12 (11.2)
Cerebral infarction	12 (11.2)
Deep vein thrombosis	4 (3.7)
Others	7 (6.5)
PT-INR	
Warfarin	2.2 ± 0.3
DOAC	1.1 ± 0.1

Characteristics of the polyps

Table 2 summarizes the characteristics of the polyps. The mean size of the polyps was 6.7 ± 3.6 mm. Approximately half were located in the right colon, and the majority (76.5%) were protruded. More than 90% (91.5%) of the polyps were adenomatous. All mucosal defects were closed by clips.

**Table 2:** Polyp characteristics. Data are presented as mean ± standard deviation or n (%).

	n = 341
Number of polyps per patient	3.6 ± 2.3
Size, mm	6.7 ± 3.6
Location	
Right colon	190 (55.7)
Left colon	120 (35.2)
Rectum	31 (9.1)
Morphology	
Pedunculated	11 (3.2)
Protruded	261 (76.5)
Superficially elevated	69 (20.2)
Histology	
Adenomatous polyp	312 (91.5)
Serrated polyp	29 (8.5)

Rates of major and minor bleeding and other adverse events

Table 3 summarizes the rates of adverse events. Major bleeding occurred in five (4.7%) patients (95% CI: 2.0%-10.5%), all of whom had delayed bleeding. Minor bleeding was observed in eight (7.5%) patients (95% CI: 3.8%-14.1%). Thromboembolism occurred in one (0.9%) patient (95% CI: 0.2%-0.5%).

**Table 3:** Rates of bleeding and other adverse events. * Active immediate bleeding did not require endoscopic management.

n = 107	Events, n	Proportion (95% CI)
Major bleeding	5	4.7% (2.0%–10.5%)
Delayed bleeding	5	-
Active immediate bleeding*	0	-
Minor bleeding	8	7.5% (3.8%–14.1%)
Thromboembolism	1	0.9% (0.2%–0.5%)

Difference in bleeding rates between warfarin and DOAC therapy

Table 4 outlines the bleeding rates associated with warfarin and DOAC use. No significant differences were found in the rates of major or minor bleeding between patients using warfarin and those using DOACs.

**Table 4:** Differences in bleeding rates between warfarin and DOACs. Data are presented as n (%). Fisher’s exact test was used to calculate the p-value. Relative risk was calculated by dividing the probability of an event occurring for warfarin by the probability of an event occurring for DOAC. DOACs: direct oral anticoagulants.

	Warfarin (n = 56)	DOACs (n = 51)	P-value	Relative risk (95% CI)
Major bleeding	2 (3.6)	3 (5.9)	0.58	0.60 (0.10-3.48)
Minor bleeding	6 (10.7)	2 (3.9)	0.27	2.73 (0.57-12.93)
All bleeding	8 (14.3)	5 (9.8)	0.56	1.45 (0.50-4.16)

Table 5 summarizes the relationship between the number of polyps and bleeding. While no significant difference was observed overall, for cases of major bleeding, the number of polyps tended to be higher in patients using warfarin (p = 0.08).

**Table 5:** Relation between number of polyps and bleeding. The number of polyps is presented as mean. DOACs: direct oral anticoagulants.

	Warfarin (n = 56)			DOACs (n = 51)		
	Major bleeding (＋)	Major bleeding (－)	P-value	Major bleeding (＋)	Major bleeding (－)	P-value
Number of polyps	7.5	3.7	0.08	2.6	3.6	0.48
	Minor bleeding (＋)	Minor bleeding (－)	P-value	Minor bleeding (＋)	Minor bleeding (－)	P-value
Number of polyps	4.1	3.8	0.72	4.5	3.5	0.37

Cases of major bleeding and thromboembolism

Table 6 presents each case of adverse events. All major bleeding incidents were managed endoscopically, with only one patient requiring a blood transfusion. Thromboembolism occurred in one patient with comorbid valvular heart disease and a history of cerebral infarction. This patient was on dual therapy with warfarin and a thienopyridine, which had been discontinued one week before EMR. The EMR procedure was completed successfully, and thienopyridine therapy was resumed two days post-EMR. However, 19 days after EMR, the patient developed a lacunar cerebral infarction. Following treatment, the patient fully recovered and was doing well at the time of this writing.

**Table 6:** Cases of major bleeding and thromboembolism. * D: descending colon; R: rectum; T: transverse colon; Ip: pedunculated; Is: protruded; IIa: superficially elevated. AE: adverse event; AF: atrial fibrillation; CI: cerebral infarction; DOAC: direct oral anticoagulant; M: male; POD: postoperative day; PT-INR: prothrombin time-international normalized ratio; TE: thromboembolism; VHD: valvular heart disease.

Age (years)/sex	Anticoagulant	Antiplatelet	Comorbidity	AE	Day of AE	Polyp characteristics associated with bleeding*	PT-INR on the day of AE	Intervention
61/M	Warfarin	－	AF	Bleeding	POD 4	R, 12 mm, Ip	2.74	Endoscopic clip
76/M	Warfarin	－	AF	Bleeding	POD 3	R, 6 mm, Is	3.47	Endoscopic clip
68/M	DOAC	－	AF	Bleeding	POD 2	T, 10 mm, Ip	1.02	Endoscopic clip
67/M	DOAC	－	AF	Bleeding	POD 9	R, 10 mm, IIa	1.17	Endoscopic clip
62/M	DOAC	－	AF, CI	Bleeding	POD 1	D, 4 mm, Is	1.62	Endoscopic clip, blood transfusion
61/M	Warfarin	Thienopyridine	VHD, CI	TE	POD 19	－	2.68	Medication

## Discussion

This study assessed the bleeding rate in patients who continued anticoagulant therapy during EMR for colorectal polyps. The overall major bleeding rate was 4.7% (95% CI: 2.0%-10.5%). The upper limit of the 95% CI was below the predefined threshold of 20%, achieving the primary endpoint.

Historically, the standard approach for patients on anticoagulants undergoing endoscopic polypectomy involved peri-procedural heparin replacement [9]. Previous studies reported significant bleeding rates (22%-24%) associated with polypectomy performed under heparin replacement therapy, even when prophylactic clip closure was employed [10,14]. A more recent study showed that the major bleeding rate following polypectomy with electrocautery, including EMR, in patients under heparin replacement therapy was also high (12%) [16].

To address the drawbacks of heparin replacement, updated guidelines now recommend continuing anticoagulants during EMR without the need for heparin replacement. However, the actual bleeding rate in patients who continue anticoagulants during EMR remains poorly understood. A prior report published before the guideline update indicated a high bleeding rate (14%) in patients continuing warfarin during polypectomy [15]. This earlier study focused on the safety of cold snare polypectomy, for which prophylactic endoscopic clips were not applied. By contrast, current practice generally involves using endoscopic clips to close mucosal defects after polypectomy in patients continuing anticoagulants [17]. Accordingly, this study incorporated endoscopic clip closure of mucosal defects as a protocol and successfully demonstrated a lower major bleeding rate in patients who continued anticoagulants during EMR.

In our study, five patients on anticoagulant monotherapy experienced major delayed bleeding. All of these patients were men, although the reason for this is unknown. Additionally, one patient receiving dual therapy with an anticoagulant and a thienopyridine developed thromboembolism after EMR. Dual therapy has been shown to increase the risk of post-EMR bleeding [18]; therefore, cessation of antiplatelet agents is generally recommended. Previous reports have not clearly established the risk of thromboembolism in patients who discontinue antithrombotic agents during polypectomy compared with those who continue their therapy [18]. In our case, however, discontinuation of thienopyridine may have contributed to the occurrence of thromboembolism.

Cold snare polypectomy without electrical current may be a preferable option for smaller polyps, but even with this technique, the delayed bleeding rate remains high in patients continuing antithrombotic agents [16,19]. Thus, careful management is required for patients on antithrombotic therapy undergoing polypectomy.

While this study demonstrates the safety of continued anticoagulants during EMR, it has several limitations. First, because this was a single-arm study, the findings should be validated through randomized controlled trials, particularly those evaluating other endoscopic techniques such as cold snare polypectomy. Second, the bleeding risk in patients on dual therapy with anticoagulants and antiplatelets was not fully assessed, necessitating further research on the management of such patients during polypectomy. Third, the lesions included in this study were <20 mm in size, which is generally recommended for resection using EMR [20]. The bleeding rate associated with EMR for larger lesions should be evaluated in future studies. Finally, although the incidence of other adverse events in patients on anticoagulants during polypectomy and the relationship between the number of resected polyps and the bleeding rate were not primary endpoints of this study, these aspects also warrant further investigation.

## Conclusions

In conclusion, the rate of major bleeding during EMR in patients who continued anticoagulants was 4.7%, which is lower than the rates reported for heparin replacement. This suggests that continued anticoagulants during EMR may be a viable and acceptable option for managing anticoagulated patients.

Further studies evaluating the impact of continuing anticoagulants during different types of endoscopic resections and exploring bleeding risks associated with various DOACs and warfarin are warranted in the future.
